# Supramolecular Surface Functionalization of Iron Oxide Nanoparticles with α-Cyclodextrin-Based Cationic Star Polymer for Magnetically-Enhanced Gene Delivery

**DOI:** 10.3390/pharmaceutics13111884

**Published:** 2021-11-06

**Authors:** Hanyi Li, Erwin Peng, Feng Zhao, Jun Li, Junmin Xue

**Affiliations:** 1 Department of Materials Science and Engineering, National University of Singapore, 7 Engineering Drive 1, Singapore 117574, Singapore; lihanyi@u.nus.edu (H.L.); a0034161@u.nus.edu (E.P.); 2 Faculty of Dentistry, National University of Singapore, 9 Lower Kent Ridge Road, Singapore 119085, Singapore; 3 Department of Biomedical Engineering, National University of Singapore, 15 Kent Ridge Crescent, Singapore 119276, Singapore; g0801506@u.nus.edu

**Keywords:** iron oxide, magnetic nanoparticles, supramolecular complexation, cyclodextrin, oligoethylenimine, gene transfection

## Abstract

Supramolecular polymers formed through host–guest complexation have inspired many interesting developments of functional materials for biological and biomedical applications. Here, we report a novel design of a non-viral gene delivery system composed of a cationic star polymer forming supramolecular complexes with the surface oleyl groups of superparamagnetic iron oxide nanoparticles (SPIONs), for magnetically enhanced delivery of DNA into mammalian cells. The cationic star polymer was synthesized by grafting multiple oligoethylenimine (OEI) chains onto an α-cyclodextrin (α-CD) core. The SPIONs were synthesized from iron(III) acetylacetonate and stabilized by hydrophobic oleic acid and oleylamine in hexane, which were characterized in terms of their size, structure, morphology, and magnetic properties. The synthesized magnetic particles were found to be superparamagnetic, making them a suitable ferrofluid for biological applications. In order to change the hydrophobic surface of the SPIONs to a hydrophilic surface with functionalities for plasmid DNA (pDNA) binding and gene delivery, a non-traditional but simple supramolecular surface modification process was used. The α-CD-OEI cationic star polymer was dissolved in water and then mixed with the SPIONs stabilized in hexane. The SPIONs were “pulled” into the water phase through the formation of supramolecular host–guest inclusion complexes between the α-CD unit and the oleyl surface of the SPIONs, while the surface of the SPIONs was changed to OEI cationic polymers. The α-CD-OEI-SPION complex could effectively bind and condense pDNA to form α-CD-OEI-SPION/pDNA polyplex nanoparticles at the size of ca. 200 nm suitable for delivery of genes into cells through endocytosis. The cytotoxicity of the α-CD-OEI-SPION complex was also found to be lower than high-molecular-weight polyethylenimine, which was widely studied previously as a standard non-viral gene vector. When gene transfection was carried out in the presence of an external magnetic field, the α-CD-OEI-SPION/pDNA polyplex nanoparticles greatly increased the gene transfection efficiency by nearly tenfold. Therefore, the study has demonstrated a facile two-in-one method to make the SPIONs water-soluble as well as functionalized for enhanced magnetofection.

## 1. Introduction

Gene delivery is the key step of gene therapy, which is the process of introducing foreign genes such as plasmid DNA (pDNA) or short interfering RNA (siRNA) into host cells [1]. For example, for cancer gene therapy, pDNA delivery makes host cells produce therapeutic proteins, while siRNA delivery temporarily down-regulates the expression of a selected gene which causes cancer development, also known as gene silencing [2,3]. Genes are delivered into cells via a vector, which serves both as a protection for the gene from degradation as well as a means to overcome cellular barriers during the delivery process. Viral vectors are genetically modified viruses, which are efficient for delivering genes but are risky due to viral-associated pathogenesis. Therefore, non-viral vectors based on synthetic materials, such as cationic polymers, have been intensively investigated in the past two decades [4]. However, non-viral vectors often have poor transfection efficiency, due to the slow diffusion process into cells. Improving the transfection efficiency of non-viral vectors has been a great area of interest [1,2,3,4,5,6,7].

Recent advances in nanotechnology integrating modern biotechnology, physical and chemical sciences have led to the development of novel non-viral gene delivery systems, which have greatly enhanced gene transfection and improved therapeutic abilities [5,7,8,9,10]. For instance, magnetic nanoparticles were studied for guided and targeted gene delivery as well as enhancing gene transfection efficiency [11,12,13,14,15,16]. With the integration of magnetic nanoparticles into gene vectors, the delivery of genes can be guided and targeted to a specific site via the use of a magnetic field at the site (Figure S1a). Furthermore, the presence of a magnetic field can greatly increase transfection efficiency [11]. The use of magnetic nanoparticles for gene transfection is also known as magnetofection (Figure S1b) [15].

However, in order for the magnetic nanoparticles to be used in gene delivery, two conditions must be fulfilled. Firstly, the nanoparticles must be water-soluble in order to be used in vivo. Secondly, nucleic acids, such as pDNA, must be able to bind to the nanoparticles in order to be delivered into cells together with the nanoparticles. Since the phosphate group of pDNA carries a negative charge, the nanoparticles must carry positive charges in order for pDNA to bind to the nanoparticles by electrostatic attraction. A possible solution is to coat the magnetic nanoparticles with cationic polymers, liposomes, or amphiphilic surfactants [17]. For example, polyethylenimine (PEI) of high molecular weight (25 kDa) was chemically conjugated onto, or been used to crosslink iron oxide nanoparticles for enhanced magnetofection [18,19,20,21,22,23,24,25,26]. Such high molecular weight PEI is non-biodegradable and its high toxicity poses a great concern with regard to its biomedical applications [27]. Furthermore, the preparation processes of nanoparticle-polymer conjugates are tedious.

**Figure 1:**
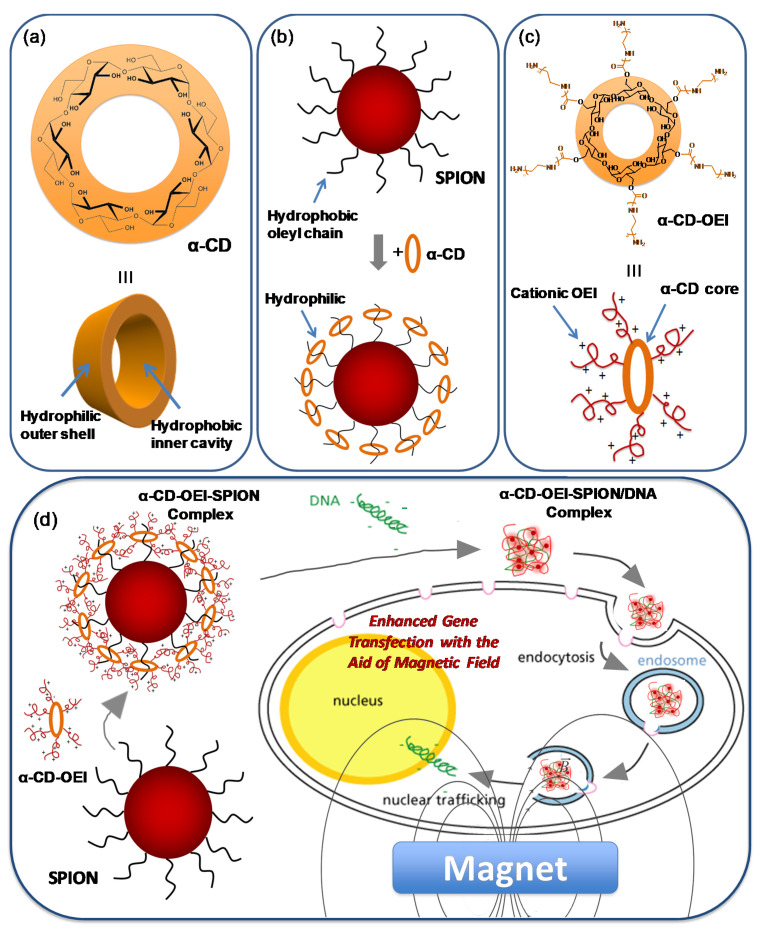
(**a**) The structure of α-CD; (**b**) Schematic representation of transfer of oleic acid-coated hydrophobic magnetic nanoparticles from organic into aqueous phase by surface modification using α-CD through inclusion complex; (**c**) Conjugating multiple arms of OEI onto an α-CD core-forming cationic star polymer α-CD-OEI as a non-viral gene vector; and (**d**) Schematic illustration of the design of the novel non-viral gene delivery system comprising magnetic nanoparticles and supramolecular polymer for enhanced magnetofection.

Cyclodextrins (CDs) are a series of natural cyclic oligosaccharides composed of 6, 7, or 8 d(+)-glucose units, also known as α-, β-, or γ-CD, respectively. The cyclic molecular structure of CDs gives a hydrophobic inner cavity with a depth of ca. 7.0 Å, and an internal diameter of ca. 4.5, 7.0, and 8.5 Å for α-, β-, and γ-CD, respectively, while the outer shell of CDs is hydrophilic due to many hydroxyl groups existing, so CDs are soluble in water (Figure 1a). The hydrophobic inner cavity of the CDs is responsible for hosting various hydrophobic guest molecules that fit into the cavity, forming supramolecular inclusion complexes that are soluble in water. The supramolecular inclusion complexes have been extensively studied as models for understanding the mechanism of molecular recognition [28,29,30] as well as building blocks for designing novel materials for various biomedical and environmental applications [31,32,33,34,35,36,37,38]. One interesting study revealed that the hydrocarbon chain of oleic acid coated on magnetic nanoparticles can be complexed by α-CD, which changes the surface property of the magnetic nanoparticles from hydrophobic to hydrophilic [39]. Hence, α-CD can be utilized for surface modification to increase the dispersity of the magnetic nanoparticles in water (Figure 1b). Another interesting study reported that conjugating multiple arms of oligoethylenimine (OEI), a low molecular weight analog of PEI, onto an α-CD core formed a cationic star polymer, α-CD-OEI, which was studied as a non-viral gene vector with low cytotoxicity and high gene transfection efficiency (Figure 1c) [27].

Being inspired by the above studies, herein we design a novel non-viral gene delivery system comprising magnetic nanoparticles and supramolecular polymer for enhanced magnetofection (Figure 1d). In this study, we synthesized superparamagnetic iron(II,III) oxide (Fe_3_O_4_) nanoparticles (SPIONs) that were stabilized by hydrophobic oleic acid and oleylamine. Thus, the SPIONs could be dispersed in a non-polar organic solvent such as hexane. We hypothesize that the α-CD-OEI star polymer can act as a host molecule like α-CD for complexing the hydrocarbon chains on the surface of the SPIONs, forming supramolecular hybrid SPIONs with hydrophilic and cationic α-CD-OEI coated on the surface. While being able to bind pDNA, the supramolecular SPIONs could act as a gene delivery vector with enhanced gene transfection efficiency with the aid of a magnetic field. Our data show that the hydrophobic SPIONs dispersed in hexane were “pulled” into the water phase by α-CD-OEI star polymer, and the resultant α-CD-OEI-SPION supramolecular complexes could bind pDNA effectively, and deliver reporter gene into cells with significantly enhanced gene transfection efficiency in the presence of the magnetic field.

## 2. Materials and Methods

### 2.1. Materials

Iron(III) acetylacetonate (99.9%), 1,2-hexadecanediol (90%), oleic acid (>99%), oleylamine (>70%), and diphenyl ether (>99%) were purchased from Sigma-Aldrich (St. Louis, MO, USA) and used as received. α-Cyclodextrin (α-CD) was supplied by Tokyo Chemical Industry (Tokyo, Japan). Branched oligoethyleneimine with molecular size of 600 Dalton (OEI-600), branched polyethyleneimine with molecular size of 25 kDa (PEI-25k), 1,1′-carbonyldiimidazole (CDI), dimethyl sulfoxide (DMSO), dimethyl ether (Et_2_O), and tetrahydrofuran (THF) were supplied by Sigma-Aldrich. Deuterium oxide (D_2_O), which was used as a solvent in NMR experiments, was supplied by Sigma-Aldrich.

Plasmid DNA pRL-CMV containing a *Renilla* luciferase reporter gene, supplied by Promega (Madison, WI, USA), was amplified in *E.* Coli and purified with a commercial kit (Endofree Plasmid Maxi Kit, Qiagen, Hilden, Germany). Luciferase kit was supplied by Promega. The 3-(4,5-dimethylthiazol-2-yl)-2,5-diphenyl tetrazolium bromide (MTT), streptomycin, and penicillin were supplied by Sigma-Aldrich. The nucleic acid sample loading buffer and SYBR safe DNA gel stain were from Bio-Rad Laboratories (Hercules, CA, USA) and Invitrogen (Waltham, MA, USA), respectively.

### 2.2. Synthesis of Superparamagnetic Fe_3_O_4_ Nanoparticles

SPIONs of 4 nm in diameter were synthesized following a procedure reported previously [17]. Iron(III) acetylacetonate (0.706 g, 2 mmol), 1,2-hexadecanediol (2.58 g, 10 mmol), oleic acid (1.70 g, 6 mmol), oleylamine (1.61 g, 6 mmol), and diphenyl ether (20 mL) were added by mass to a three-neck round-bottom flask. The reaction mixture was set to stir in a heating mantle by a magnetic stirrer. A constant flow of nitrogen gas was maintained through the flask during synthesis. The mixture was heated to 100 °C and maintained for 15 min. Next, the temperature was raised to 200 °C and maintained for 30 min. Then, the mixture was heated at 265 °C to reflux for another 30 min. After which, the heating mantle was turned off for natural cooling to room temperature, during which stirring and nitrogen flow were still maintained.

The black-brown mixture was added to large amounts of ethanol (ca. 40 mL) to precipitate the SPIONs. The resultant mixture was then centrifuged at 10,000× *g* rpm for 10 min to separate the black solid material. The supernatant was discarded. Sufficient hexane was added dropwise to dissolve the black material, after which the solution was treated in an ultrasonic bath for 10 min to allow for better dissolution. The solution was centrifuged at 6000× *g* rpm for 10 min to separate any impurities. The supernatant was extracted and the residue was discarded. This process of precipitation and dissolution of SPIONs were repeated several times. The SPIONs were finally stored in hexane at 50 mg/mL after determining the concentration by finding the mass of SPIONs in a known volume of solution.

### 2.3. Synthesis of a-CD-OEI Star Polymer

The α-CD-OEI star polymer (average arm number per CD: 5.2) was synthesized following our previously reported method [27]. Specifically, a 30 mL DMSO solution of CDI (4.86 g, 30 mmol) was added dropwise into a solution of 40 mL DMSO solution of α-CD (0.97 g, 1.0 mmol) over a period of 6 h. After stirring for 20 h under nitrogen atmosphere at room temperature, the solution was added slowly into Et_2_O/THF (400/200 mL), followed by centrifugation to collect the white precipitate. The precipitate was washed with Et_2_O/THF (20/10 mL) twice and dissolved in 40 mL of anhydrous DMSO. This solution was added dropwise into a DMSO solution (40 mL) containing OEI-600 (12.6 g, 21 mmol) over a period of 3 h, and then stirred under nitrogen atmosphere at room temperature for 24 h. Purification was carried out by dialysis membrane (MWCO: 2000) in deionized water over 5 days, followed by freeze-drying to give α-CD-OEI star polymer product with a yield of 21%. ^1^H NMR data (400 MHz, D_2_O): *δ*5.11 (s, broad, 6 protons, H_1_ of α-CD), 3.46–4.60 (m, broad, 36 protons, H_2_, H_3_, H_4_, H_5_, and H_6_ of α-CD), 3.00–3.46 (m, broad, 10 protons, -CONCH_2_- of OEI-600), 2.67 (m, 264 protons, -NCH_2_- of OEI-600).

### 2.4. Characterization

Transmission electron microscopy (TEM) was conducted using the JEOL 100CX 2010F (JEOL, Tokyo, Japan) with an accelerating voltage of 200 kV. Samples in hexane were dripped onto carbon-coated copper grids followed by air drying on an absorbent filter sheet. Images to investigate the morphology of the SPIONs were obtained.

X-ray diffractometry (XRD) was conducted on a Bruker AXS D8 Advance Diffractometer System (Bruker, Billerica, MA, USA) for studying the crystalline phase of SPIONs. Measurements were conducted with the following conditions: a Cu K_α_ radiation (λ = 1.5418 Å) source operated at 40 kV and 40 mA with a step size of 0.05° and a time per step of 5 s.

Particle size of SPIONs was analyzed using dynamic light scattering (Zetasizer Nano-ZS, Malvern Instruments, Malvern, UK). SPIONs were dissolved in 1.0 mL of hexane at a concentration of ca. 5 mg mL^−1^ in a glass sizing cuvette. The sample was equilibrated at 25 °C before measurements were taken. The measurements were made with a laser light wavelength of 633 nm at a 173° scattering angle. Two measurements were taken.

Magnetic moments of SPIONs were measured using a vibrating sample magnetometer (LakeShore Model 7407 VSM, Westerville, OH, USA) at 300 K. A sample of 5 mg of dried powder SPION was wrapped securely in non-magnetic aluminum foil and folded into a size of approximately 5 mm^2^. The sample was first saturated in a 20 kG magnetic field and then cycled from 20 kG to −20 kG and back again to 20 kG at room temperature to obtain a hysteresis curve.

The ^1^H NMR spectra were obtained on a Bruker AV-400 NMR spectrometer (Bruker, Billerica, MA, USA) at 400.1 MHz at room temperature. The measurements were conducted with an acquisition time of 3.2 s, a pulse repetition time of 2.0 s, a pulse width of 30°, a spectral width of 5208 Hz, and 32 K data points. The solvent peak (δ = 4.70 ppm for D_2_O) was used as the chemical shift reference.

### 2.5. Formation of α-CD-OEI-SPION Complex

The α-CD-OEI polymer (45 mg) was dissolved in water (2.0 mL). SPIONs were diluted using hexane into 0.5 mg/mL. In a glass bottle, 2.0 mL of α-CD-OEI polymer aqueous solution and 2.0 mL of SPION colloidal solution were added, forming separate layers of water and hexane due to the difference in density and their immiscibility. The top layer of hexane would appear yellow-brown in color due to the color of the SPIONs; the bottom layer would be colorless. The mixture was then magnetically stirred overnight. In the end, the top hexane layer appeared colorless while the bottom water layer appeared yellowish, indicating that the SPIONs had been “pulled” into the water layer by the polymer. The bottom water layer containing the α-CD-OEI-SPION complexes was then extracted and freeze-dried (yield: 44.6 mg).

### 2.6. Cells and Media

The breast cancer cell line MCF-7 was purchased from the American Type Culture Collection (ATCC, Rockville, MD, USA) and maintained in Dulbecco’s Modified Eagle’s Medium (DMEM) supplemented with 10% heat-inactivated fetal bovine serum (FBS), 100 units/mg penicillin, 100 mg/mL streptomycin in a humidified incubator with 5% CO_2_ at 37 °C.

### 2.7. Cell Viability Assay

The cell viability was assessed by MTT assay [40]. The breast cancer cell MCF-7 was used for cell viability assay. Cells were cultured in Dulbeccos Modified Eagles Medium (DMEM) (Thermo Fisher Scientific, Waltham, MA, USA). To the DMEM was added 10% in volume of fetal bovine serum to increase growth factors of the cells, and 1% in volume of the antibiotics penicillin and streptomycin. Cells were cultured for 24 h in an incubator set at a temperature of 37 °C, carbon dioxide injection of 5.0% and relative humidity of 95%. After determining the cell count, 1 × 10^4^ cells in 100 µL DMEM were seeded into each well on a 96-well plate. The cells were cultured for 24 h.

The polymer α-CD-OEI and its SPION complex were diluted to a series of concentrations ranging from 6.25 µg mL^−1^ to 100 µg mL^−1^. Polyethylenimine (PEI) was used as a control. After disposing of the existing medium and adding equal volume of fresh DMEM to the cell cultures, 20 µL of each PEI, α-CD-OEI, or α-CD-OEI-SPION complex was added to each cell culture well. The cell cultures were then incubated under the same conditions for 20 h.

At the end of 20 h, 10 µL of sterile-filtered MTT stock solution (5 mg mL^−1^) were added to each well, reaching a final MTT concentration of 0.5 mg mL^−1^. The cell cultures were incubated again for 4 h. Then, unreacted dye was discarded by aspiration. The formazan crystals were dissolved in 100 µL DMSO and absorbance was measured at a wavelength of 570 nm on the FLUOstar Optima microplate reader. The ratio, in percentage, of the cell growth in relation to control cells cultured in medium without α-CD-OEI or α-CD-OEI-SPION complex was calculated based on the cell concentrations.

### 2.8. Formation of α-CD-OEI-SPION/pDNA Polyplex Nanoparticles

A gel retardation assay was conducted to determine the DNA binding ability of the α-CD-OEI-SPION complex at different N/P ratios, following a gel electrophoresis experiment procedure reported previously [41]. The sample solutions (polymers or α-CD-OEI-SPION complex) were diluted with distilled water to give various nitrogen concentrations. Then a pDNA solution at 0.1 mg/mL was mixed with an equal volume of these solutions to prepare 10 µL of polymer/pDNA or α-CD-OEI-SPION/pDNA polyplex at different N/P ratios ranging from 0 to 10. Each sample was shaken for 10 s and left to stand for 30 min at room temperature. Subsequently, 9 µL of each polyplex solution was mixed with 2 µL of nucleic acid sample loading buffer. The mixture was loaded onto 0.8% agarose gel containing 10 µL of SYBR safe DNA gel stain. Gel electrophoresis was carried out in TAE buffer containing 40 mM Tris-acetate and 1 mM EDTA at 100 V for 30 min in a Sub-Cell system (Bio-Rad Laboratories). Finally, DNA bands were visualized with a UV lamp using a GelDoc system (Synoptics Ltd., Cambridge, UK).

Particle size and zeta potential of the polyplex nanoparticles formed by α-CD-OEI-SPION and pDNA were analyzed using dynamic light scattering (Zetasizer Nano-ZS, Malvern). α-CD-OEI-SPION was mixed with 3 µg of pDNA (0.1 mg/mL) at N/P ratios of 20 to 60 with intervals of 10, shaken for 10 s, and left to stand for 30 min at room temperature. The resultant sample solutions were diluted to 1.0 mL using water. Samples were placed in a disposable sizing cuvette for sizing measurements and a disposable zeta cell for zeta potential measurements. The samples were equilibrated at 25 °C before measurements were taken. Measurements were made with a laser light wavelength of 633 nm at a 173° scattering angle. Two size measurements and three zeta potential measurements were taken.

### 2.9. In Vitro Gene Transfection Efficiency

The breast cancer cell MCF-7 was used for gene transfection [42]. Cells were cultured in Dulbeccos Modified Eagles Medium (DMEM) (Thermo Scientific). To the DMEM was added 10% in volume of fetal bovine serum to increase growth factors of the cells, and 1% in volume of the antibiotics penicillin and streptomycin. Cells were cultured for 24 h in an incubator set at a temperature of 37 °C, carbon dioxide injection of 5% and relative humidity of 95%. After determining the cell count, 4 × 10^4^ cells in 400 µL DMEM were seeded into each well on a 24-well plate. The cells were cultured for 24 h.

The polymer α-CD-OEI or α-CD-OEI-SPION complex were mixed with pDNA at nitrogen-to-phosphate ratios (N/P ratios) of 20 to 60 with intervals of 10, shaken for 10 s, and left to stand for 30 min at room temperature. PEI was used as a control. After discarding the existing medium and adding equal volume of fresh DMEM to the cell cultures, 20 µL of PEI/pDNA, α-CD-OEI/pDNA, or α-CD-OEI-SPION/pDNA polyplexes, containing 1 µg of pDNA pRL-CMV, was added to each cell culture well. Two groups of culture plates were prepared. The first group underwent transfection in the presence of an external magnetic field. The cell culture plates were placed on a magnet (ExpressMag^®^ Super Magnetic Plate, purchased from Sigma-Aldrich) during incubation for 1.5 h. The second group underwent transfection for the same time period but without a magnet. After transfection, the medium was disposed and 400 µL fresh DMEM was added. The cell cultures were then incubated under the same conditions for 24 h to allow for the expression of the gene and the biosynthesis of proteins. Triplicates of each sample were conducted within each group.

At the end of incubation, the medium was discarded. The cell lines were washed twice with phosphate-buffered saline (PBS) and lysed with 100 µL of lysis buffer (Promega) over 1 h of shaking of the plates. A volume of 10 µL of lysis buffer was then extracted into an opaque 96-well plate for luciferase assay. The plate was placed in the Berthold Centro LB 960 microplate luminometer, and 50 µL of luciferase assay buffer (Promega) was injected automatically before the plate was shaken for 10 s, left to stand for 10 s, and then the luminesce measurement was taken. A volume of 10 µL of lysis buffer was extracted as well into a 96-well plate for protein assay. To each well, 240 µL of Coomassie Plus protein assay reagent (Pierce, Waltham, MA, USA) was added before its absorbance was measured at a wavelength of 595 nm on the BMG Labtech FLUOstar Optima microplate reader (Ortenberg, Germany). From these data, the relative light units per unit mass of protein (RLU mg^−1^ protein) were determined, which represented the efficiency of the gene transfection process.

## 3. Results and Discussion

### 3.1. Synthesis and Characterization of SPIONs

**Figure 2:**
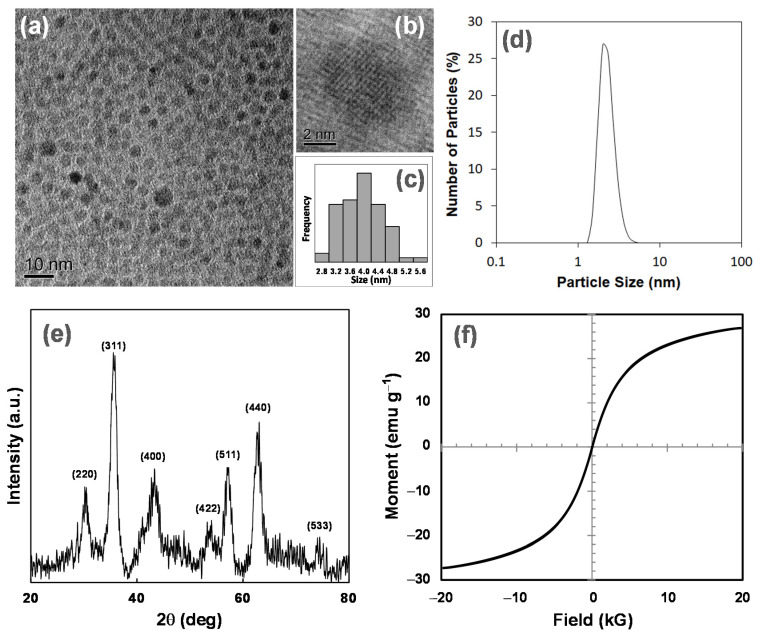
(**a**) TEM bright field image of the SPIONs; (**b**) High-resolution TEM image of an isolated SPION; (**c**) Bar chart showing the number size distribution profile obtained from image a, mean diameter: 3.9 ± 0.6 nm; (**d**) Particle size distribution of the SPIONs measured by DLS; (**e**) X-ray diffraction spectrum of the SPIONs; and (**f**) Magnetic profile of 4 nm SPIONs.

Superparamagnetic iron oxide nanoparticles (SPIONs) of 4 nm in size were synthesized with iron (III) acetylacetonate as the precursor according to a method reported (Figure S2) [17]. The particles synthesized were completely dispersed in hexane, a typical non-polar solvent. We obtained TEM images to study the morphology of the SPIONs. Figure 2a shows that the particles synthesized had a very narrow size distribution. The particles were well-dispersed with no apparent agglomeration. Figure 2b shows a distinct isolated particle that is approximately 4.5 nm in size. The core diameters of the nanoparticles in Figure 2a were measured against the scale bar, and the results are shown in Figure 2c. The mean diameter of the particles was found to be 3.9 nm. The results indicate that the desired narrowly dispersed 4 nm particles were obtained via this method. The narrow dispersity of the SPIONs was further supported by the DLS results as shown in Figure 2d, where only a single and sharp peak was observed at a size of approximately 4.0 nm.

XRD was used to verify the crystallographic structure of the SPIONs synthesized. The XRD spectrum shown in Figure 2e displays characteristic peaks, which were referenced to the Fe_3_O_4_ magnetite phase in the PDF database (PDF #65-3107). The peaks showed a match to the PDF database upon comparison, and the *hkl* indexes of the peaks are labeled in Figure 2e. This confirms that the SPIONs synthesized mainly composed of the Fe_3_O_4_ magnetite phase.

The magnetic property of the SPIONs was characterized by vibrating sample magnetometer (VSM) measurement at 300 K, and the magnetic profile of the SPIONs is shown in Figure 2f. The saturation magnetization (M_s_) was seen to be 26.9 emu g^−1^. The profile passes through the origin of the graph, which implies that magnetization in an absence of an external field is zero at room temperature. There is also an absence of a hysteresis loop. The results prove that the SPIONs synthesized are effectively superparamagnetic.

This is an important characteristic for dispersed SPIONs to exhibit properties of ferrofluids, which would be helpful in biological applications. The ferrofluid can be directed to a specific area in the body after being injected intravenously. At the same time, superparamagnetic ferrofluids are safer and more suitable for biological applications as compared to conventional ferromagnetic ferrofluids. Ferromagnetic ferrofluids may retain magnetization after exposure to magnetic field for a long period of time. In the absence of an external magnetic field, there may be the risk of agglomeration of ferromagnetic particles if there is remnant magnetization and the surfactant is unstable and insufficient. In such situations, excretion of the particles may be difficult or even cause complications in the circulatory system. Superparamagnetic particles, which do not retain magnetization, do not have such potential problems.

### 3.2. Formation of α-CD-OEI-SPION Complex

The surface of the SPIONs was coated with hydrophobic oleyl hydrocarbon chains, so the SPIONs were well dispersed in nonpolar solvents such as hexane. It is thought that oleyl hydrocarbon chains can fit well into the hydrophobic inner cavity of α-CD because of the hydrophobic interaction as well as the good match in size [43]. It was reported that α-CD could form inclusion complexes with the hydrocarbon chains and then change the surface of SPIONs from hydrophobic into hydrophilic (Figure 1b) [39]. However, the reported α-CD-stabilized SPIONs had no functions for gene carriers. Herein, we hypothesize that the α-CD core of the star polymer α-CD-OEI would be able to form inclusion complexes with the oleyl hydrocarbon chains of the SPIONs in a similar manner (Figure 1d). Since multiple OEI chains are grafted to the α-CD core in α-CD-OEI, the surface of the α-CD-OEI-SPION complex will be covered with many cationic OEI chains, so the α-CD-OEI-SPION complex should be able to bind and condense DNA to form DNA nanoparticles, in which the SPIONs are encapsulated. Therefore, the α-CD-OEI-SPION/pDNA nanoparticles may be suitable for efficient cellular internalization, and the cellular internalization could be aided by magnetic field to lead to enhanced gene transfection.

**Figure 3:**
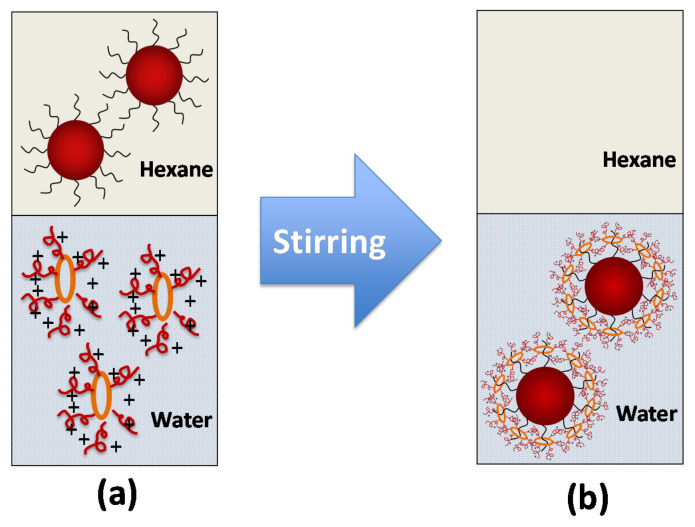
Schematic illustrations of the immiscible layers of hexane and water before (**a**) and after (**b**) the process transferring oleic acid stabilized SPIONs from organic into aqueous phase by surface modification and complexation with α-CD-OEI. The top layer is the hexane phase, and the bottom layer is the aqueous phase.

In this study, the star polymer α-CD-OEI was synthesized following our previously reported method [27], and the average arm number per α-CD core was 5.2, which was determined by a ^1^H NMR spectrum. The formation of the α-CD-OEI-SPION complex was conducted by the vigorous stirring of an immiscible mixture of a water phase with α-CD-OEI dissolved and a hexane phase with SPIONs dispersed (Figure 3). The process of phase transfer from the organic phase to the aqueous phase could be observed by the change of colors of the two immiscible layers in the mixture. The mixture initially started with a colorless α-CD-OEI aqueous solution layer at the bottom and a yellow-brown hexane layer at the top due to the dispersed SPIONs (Figure S3a). At the end of the phase transfer, the bottom aqueous layer became yellowish, while the intensity of the yellow-brown in the top hexane layer has decreased and finally almost disappeared (Figure S3b).

The hexane and water layers of the resultant mixture were separated and dried. The remaining solid in hexane (SPIONs) was a trace, while the solid in the water had a significant increase in mass. This indicates that there indeed was a transfer of SPIONs from the hexane layer to the aqueous layer. When dried SPIONs were added to any polar solvent such as ethanol or water, the particles were precipitated immediately in the form of a brown solid due to the hydrophobic surface of the particles. However, after phase change, the aqueous layer remained with no sign of precipitation of any solid. A control experiment was also carried out with distilled water replacing the α-CD-OEI aqueous solution. This experiment showed no change in the colorization of the hexane and water layers. Thus, it is sufficient to conclude that the presence of α-CD-OEI allowed the SPIONs to be dispersible in water due to the formation of the α-CD-OEI-SPION complex.

### 3.3. Cytotoxicity

**Figure 4:**
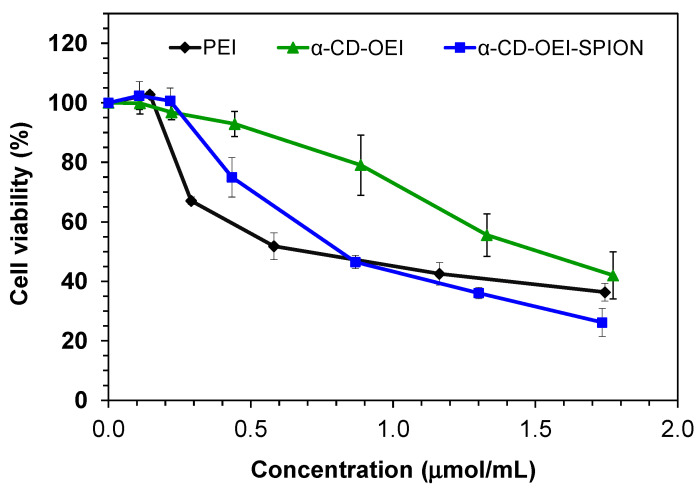
Cell viability of MCF-7 cells in the presence of PEI, α-CD-OEI, and α-CD-OEI-SPION at various molar concentrations in terms of the ethylenimine (-CH_2_CH_2_NH-) units contained in each carrier.

It is important to consider cytotoxicity for materials used in gene delivery. The main reason for the increased toxicity of high molecular weight PEI is thought to be the aggregation and adherence of PEI on the cell surface [44]. The introduction of cyclodextrin in PEI or OEI can decrease cytotoxicity [45]. We reported that α-CD-OEI has lower cytotoxicity than high molecular weight PEI [27]. This was also proven as seen in Figure 4, showing that α-CD-OEI was much less cytotoxic in MCF-7 cells. The cell viability for PEI dropped to 50% at the concentration of 0.82 µmol mL^−1^, while that for α-CD-OEI dropped to 50% at the concentration of 1.62 µmol mL^−1^. For α-CD-OEI-SPION, with the addition of SPIONs to α-CD-OEI, cytotoxicity increased. The cell viability decreased to 50% at the concentration of 0.84 µmol mL^−1^. Likely this is because α-CD-OEI-SPION is in the form of nanoparticles. α-CD-OEI is a water-soluble polymer with relatively low molecular weight (about 4090 Da, much lower than PEI 25 kDa), which is taken by cells mainly through diffusion. In contrast, α-CD-OEI-SPION nanoparticles can be taken by cells through endocytosis, which is considered more efficient cellular internalization, then causing higher cytotoxicity than α-CD-OEI.

### 3.4. Formation of the α-CD-OEI-SPION/pDNA Polyplex

**Figure 5:**
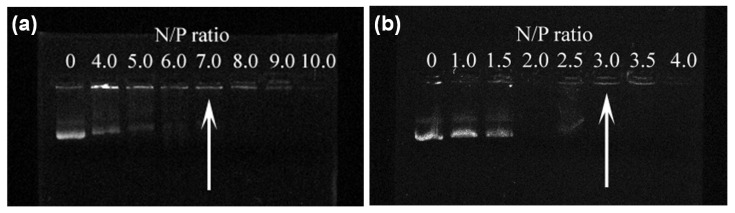
Results of the gel retardation assay for pDNA in the polyplexes formed with (**a**) α-CD-OEI and (**b**) α-CD-OEI-SPION at different N/P ratios. The arrow indicates that pDNA was fully retarded by formation of polyplexes.

The ability of the α-CD-OEI-SPION complex to bind pDNA was examined by their electrophoretic mobility on an agarose gel at various nitrogen-to-phosphate ratios (N/P ratios). As seen in Figure 5, α-CD-OEI showed effective binding with pDNA at an N/P ratio of 7.0 (Figure 5a), while the α-CD-OEI-SPION complex could bind pDNA more effectively, retarding pDNA at a lower N/P ratio of 3.0 (Figure 5b).

The increased DNA binding ability is possibly due to the fact that the formed α-CD-OEI-SPION complex involved many α-CD-OEI molecules threaded onto the hydrocarbon chains on the surface of a SPION, resulting in a particle with many OEI segments integrated into a larger molecular entity. When relatively small α-CD-OEI molecules bind with pDNA, the binding is solely the result of electrostatic attraction between separate α-CD-OEI molecules and pDNA. In the case of pDNA binding with the α-CD-OEI-SPION complex, the integrated α-CD-OEI molecules around the particle will result in stronger interaction with the pDNA molecules. This will result in stronger binding of the α-CD-OEI-SPION complex with pDNA molecules as compared to solely α-CD-OEI molecules.

**Figure 6:**
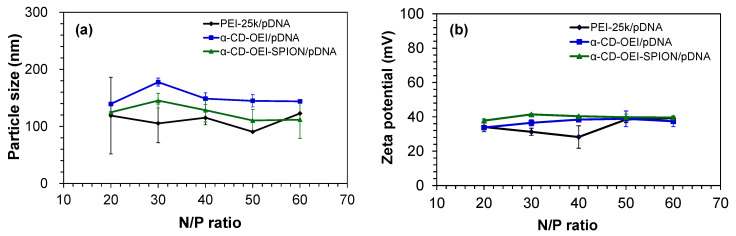
Results of particle size (**a**) and zeta potential (**b**) measurements for polyplexes formed by pDNA with PEI (25kDa), α-CD-OEI, and α-CD-OEI-SPION at various N/P ratios.

The particle size and surface charge of α-CD-OEI-SPION/pDNA polyplexes are important parameters in gene delivery, which may influence the particles’ blood circulation time, cell internalization mechanism, and bioavailability [46,47]. Particle sizes of pDNA polyplexes of α-CD-OEI and α-CD-OEI-SPION, with reference to PEI, are plotted in Figure 6a. The particle sizes range approximately from 100 to 200 nm, which is considered suitable for cell internalization and gene delivery. Comparing α-CD-OEI-SPION/pDNA polyplex with the initial 4 nm SPION, there was a large increase in size. The reason is thought to be the aggregation of multiple α-CD-OEI-SPION complexes with pDNA molecules, largely increasing the overall size of the α-CD-OEI-SPION/pDNA polyplex nanoparticles. Nevertheless, the size of the polyplex particles ranging from 100 to 200 nm is very suitable for cellular uptake [48].

The zeta potential graphs are shown in Figure 6b, indicating that all the pDNA polyplexes with PEI, α-CD-OEI and α-CD-OEI-SPION carried a positive surface charge at the N/P ratios from 20 to 60, ranging from about 30 to 40 mV. After binding pDNA, the complexes still carried positive surface charges due to the high N/P ratios used which gave excess nitrogen (amine) groups. Positively charged polyplexes tend to bind to the cell membrane and may be easily taken up by cells through endocytosis, one of the main mechanisms of cellular uptake.

### 3.5. In Vitro Gene Transfection and Luciferase Assay

**Figure 7:**
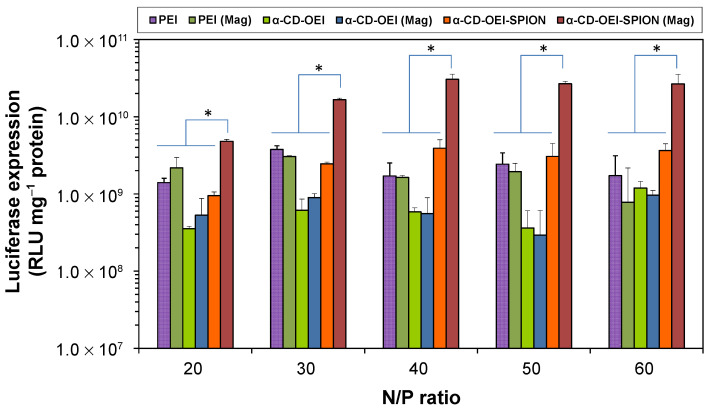
Results of luciferase assay for pDNA polyplexe nanoparticles formed with PEI (25 kDa), α-CD-OEI, and α-CD-OEI-SPION at various N/P ratios in MCF-7 cells, showing the different in vitro gene transfection efficiency without and with Magnetic Plate (Mag). Data represent mean ± standard deviation (* *p* < 0.05, *n* = 3).

When negatively charged pDNA molecules encounter cationic polymer chains in water, the electrostatic interaction will cause the phase separation of pDNA and the polymer, leading to the formation of condensed pDNA/polymer polyplex as colloidal nanoparticles, which are suitable for cellular internalization through endocytosis and/or other pathways. To demonstrate the gene transfection efficiency of the newly developed α-CD-OEI-SPION supramolecular magnetic gene vector, luciferase-encoded pDNA pRL-CMV was used to form α-CD-OEI-SPION/pDNA polyplex nanoparticles, and the in vitro luciferase marker gene transfection was carried out using MCF-7 cancer cell line. Results on gene transfection efficiencies using PEI (25 kDa), α-CD-OEI, and α-CD-OEI-SPION without and with external magnetic field are shown in Figure 7. For gene transfection mediated by PEI and α-CD-OEI at all N/P ratios tested, the gene transfection efficiency without or with the magnetic field was nearly the same. As expected, the transfection efficiency of PEI and α-CD-OEI were not affected by the presence of a magnetic field due to the absence of magnetic materials. In contrast, when the gene transfection mediated by α-CD-OEI-SPION was carried out under an external magnetic field, it was observed that the transfection efficiency showed a significant increase by approximately ten folds as compared to that without a magnetic field. Furthermore, the gene transfection efficiency of α-CD-OEI-SPION/pDNA polyplex under magnetic field was much higher than all other control groups in this study, including the high molecular weight PEI 25 kDa. Therefore, the newly developed α-CD-OEI-SPION gene vector has shown a great magnetofection effect as hypothesized.

The gene transfection results demonstrate that the presence of SPIONs in the α-CD-OEI-SPION gene vector greatly enhances the efficiency of gene delivery under an external magnetic field. A magnetic field attracts α-CD-OEI-SPION/pDNA polyplex nanoparticles to the cell membrane, increasing the nanoparticle concentration at the cell membrane, and speeding up the sedimentation of the nanoparticles into the cells, while in the absence of a magnetic field the gene delivery merely relies on diffusion of the polyplex nanoparticles before approaching cells. The findings are in good agreement with previous reports for other magnetic gene vectors containing iron oxide magnetic nanoparticles and PEI that were fabricated by different methods [18,19,20,21,22,23,24,25,26]. There are also reports on SPION-encapsulated polymer nanovehicles that show enhanced cellular uptake under external magnetic fields [49]. Here, the use of the simple supramolecular complexation process to convert the hydrophobic SPIONs into hydrophilic ones with many cationic OEI chains coated on the surface has provided a facile two-in-one method to make the SPIONs water-soluble as well as functionalized for enhanced magnetofection.

## 4. Conclusions

The oleyl-coated hydrophobic SPIONs were synthesized from iron(III) acetylacetonate. The SPIONs dispersed in hexane were treated with an α-CD-OEI star polymer dissolved in water, and the SPIONs were transferred from hexane into the water phase due to the surface of the SPIONs being converted from hydrophobic to hydrophilic through supramolecular complexation between an α-CD unit of the α-CD-OEI star polymer and the oleyl chains on the nanoparticle surface. The α-CD-OEI-SPION complex could efficiently bind and condense pDNA to form nano-sized DNA polyplex nanoparticles suitable for cellular internalization. The gene transfection mediated by the α-CD-OEI-SPION complex was greatly enhanced by the external magnetic field. The use of an α-CD-OEI star polymer is a novel method to solubilize hydrophobic SPIONs in water, and impart the α-CD-OEI-SPION complex to bind and condense pDNA at the same time, enabling the complex to be a non-viral vector for magnetically enhanced and targeted gene delivery. Therefore, this work has demonstrated a proof-of-concept study of the novel surface functionalization of SPIONs through supramolecular inclusion complexation using our previously developed α-CD-OEI cationic star polymer, as a very convenient two-in-one process to turn the hydrophobic SPIONs into a water-soluble gene carrier with magnetically enhanced and targeted gene transfection. If applied in vivo, the magnetic field can attract the α-CD-OEI-SPION/pDNA polyplex nanoparticles to accumulate along the specific target tissues or organs, encouraging greater cellular uptake of pDNA for efficient gene therapy. We believe that this work will stimulate future studies on its applications for magnetically enhanced and targeted delivery of therapeutic genes (DNA or RNA) and even chemotherapeutic drugs. It is also interesting to further study the mechanisms on how the gene transfection is enhanced and how the SPIONs will impact the cytotoxicity of the delivery systems.

## Data Availability

Available from the corresponding authors upon request.

## Supplementary Materials

The following are available online at https://www.mdpi.com/article/10.3390/pharmaceutics13111884/s1, Figure S1: Schematic illustrations for (a) magnetic nanoparticle guided and targeted gene delivery in-vivo, and (b) magnetic nanoparticle mediated gene delivery in-vitro. The vector is attached to magnetic nanoparticles, which are added to the cell culture. The magnetic field gradient pulls the particles towards the magnetic field source, increasing the sedimentation rate of the particle/gene complex, Figure S2: (a) Synthesis scheme of 4 nm superparamagnetic iron oxide nanoparticle (SPION); and (b) The experimental setup for the synthesis, Figure S3: Optical photographs of the immiscible layers of hexane and water before (a) and after (b) the process transferring oleic acid stabilized SPIONs from organic into aqueous phase by surface modification and complexation with α-CD-OEI. The top layer is the hexane phase, and the bottom layer is the aqueous phase.
