# Supplementary material for: Supramolecular Surface Functionalization of Iron Oxide Nanoparticles with α-Cyclodextrin-Based Cationic Star Polymer for Magnetically-Enhanced Gene Delivery
Source: Pharmaceutics. 2021 Nov 6;13(11):1884. doi: 10.3390/pharmaceutics13111884 (PMC8624969; doi:10.3390/pharmaceutics13111884)
Supplement: Supplementary file 1 [file pharmaceutics-13-01884-s001.zip › pharmaceutics-1422285-supplementary.pdf]

pharmaceutics

MDPI

Supplementary Materials: Supramolecular Surface Functionalization of Iron Oxide Nanoparticles with α-Cyclodextrin-Based Cationic Star Polymer for Magnetically-Enhanced Gene Delivery

Hanyi Li, Erwin Peng, Feng Zhao, Jun Li and Junmin Xue

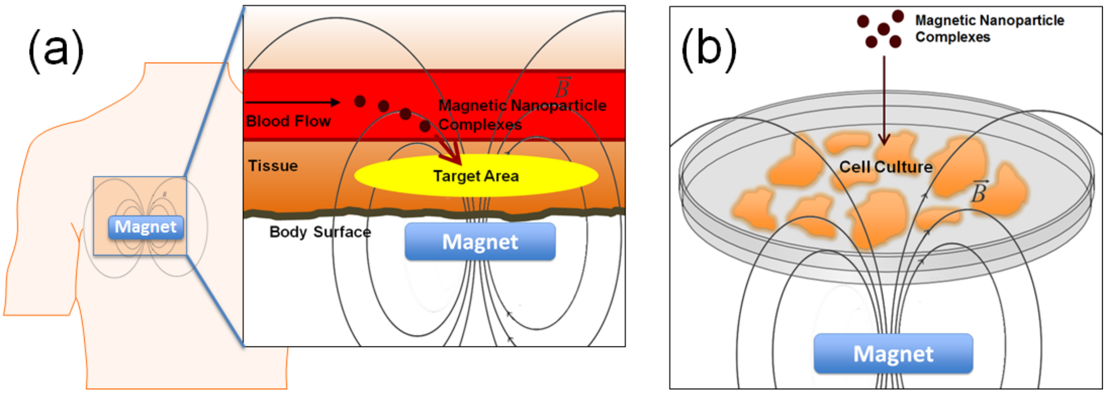

Figure S1. Schematic illustrations for (**a**) magnetic nanoparticle guided and targeted gene delivery *in-vivo*, and (**b**) magnetic nanoparticle mediated gene delivery *in-vitro*. The vector is attached to magnetic nanoparticles, which are added to the cell culture. The magnetic field gradient pulls the particles towards the magnetic field source, increasing the sedimentation rate of the particle/gene complex [1].

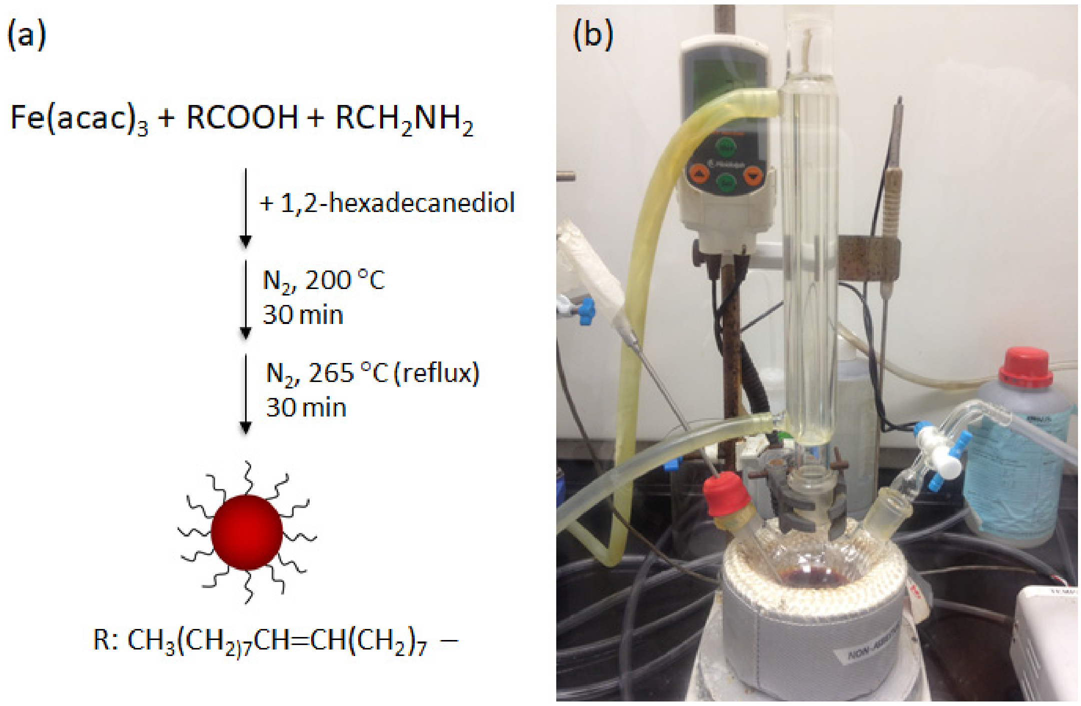

Figure S2. (**a**) Synthesis scheme of 4 nm superparamagnetic iron oxide nanoparticle (SPION); and (**b**) The experimental setup for the synthesis.

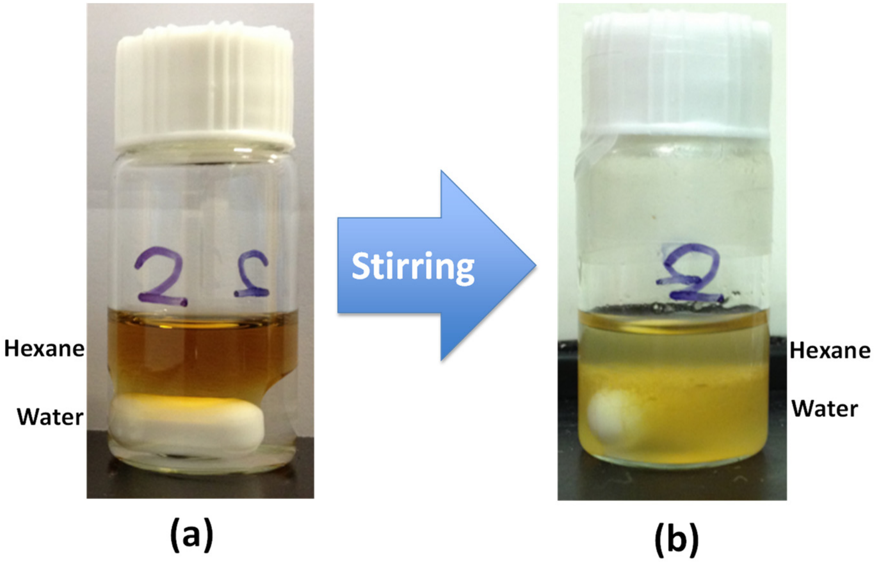

Figure S3. Optical photographs of the immiscible layers of hexane and water before (**a**) and after (**b**) the process transferring oleic acid stabilized SPIONs from organic into aqueous phase by surface modification and complexation with α-CD-OEI. The top layer is the hexane phase, and the bottom layer is the aqueous phase.

Reference

1. Dobson, J., Gene therapy progress and prospects: magnetic nanoparticle-based gene delivery. Gene Therapy **2006**, *13*, (4), 283–287.
